# P-554. The Validity of UV Light Devices’ Advertised Kill Rates

**DOI:** 10.1093/ofid/ofaf695.769

**Published:** 2026-01-11

**Authors:** Anya Jinadatha

**Affiliations:** Belton High School, Belton, TX

## Abstract

**Background:**

Food borne illnesses are a major concern. According to the Centers for Disease Control and Prevention (CDC), each year, over 48 million people suffer from food borne illnesses. Ultraviolet light has emerged as a solution for tackling relevant food safety concerns due to its proven effectiveness in the disinfection of grocery and residential kitchen surfaces through the deactivation of viruses, bacteria, fungi, yeasts, and molds. To promote their products, companies selling handheld UV light devices on platforms like Amazon make claims about the efficiency of their devices to disinfect grocery and kitchen surfaces. This study aimed to determine whether the disinfection efficacy of the handheld UV disinfection devices matched manufacturers’ claims.

**Figure:**
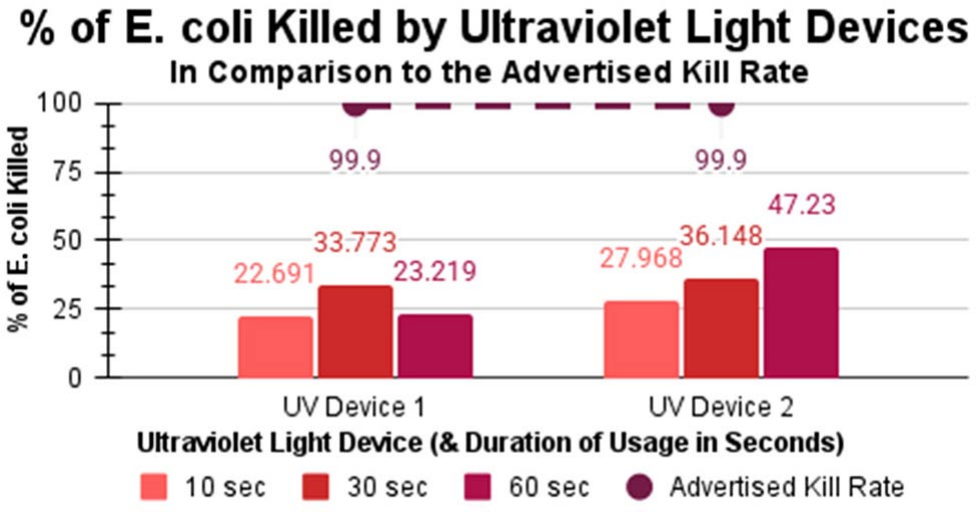

UV Device 1 caused slight decline in E. coli, with kill rates of 22.69% at 10 seconds, 33.77% at 30 seconds, and 23.22% at 60 seconds. Similarly UV Device 2 caused minimal decline in E. coli with kill rates of 27.97% at 10 seconds, 36.15% at 30 seconds, and 47.23% at 60 seconds. Notably, neither device, regardless of the treatment duration, succeeded in reaching the advertised kill rate of 99.9%. As further explained in the conclusions, these results hold many implications, particularly in food safety, for users of these UV devices under the misconception that they kill harmful bacteria such as E. coli.

**Figure:**
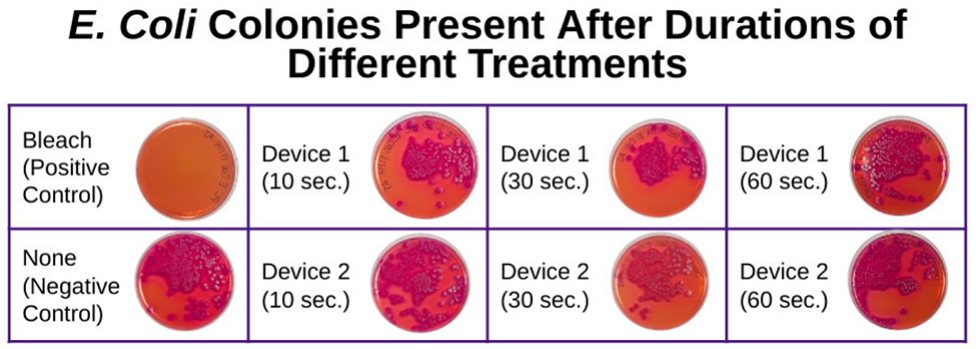

**Methods:**

The 2 top-reviewed handheld UV disinfection devices from Amazon were tested on Escherichia coli (E. coli) to determine their survival rate after 10, 30, and 60 seconds of irradiation. The kill rate for each UV device was calculated by dividing the E. coli killed due to irradiation by the negative control, then multiplied by 100.

**Figure:**
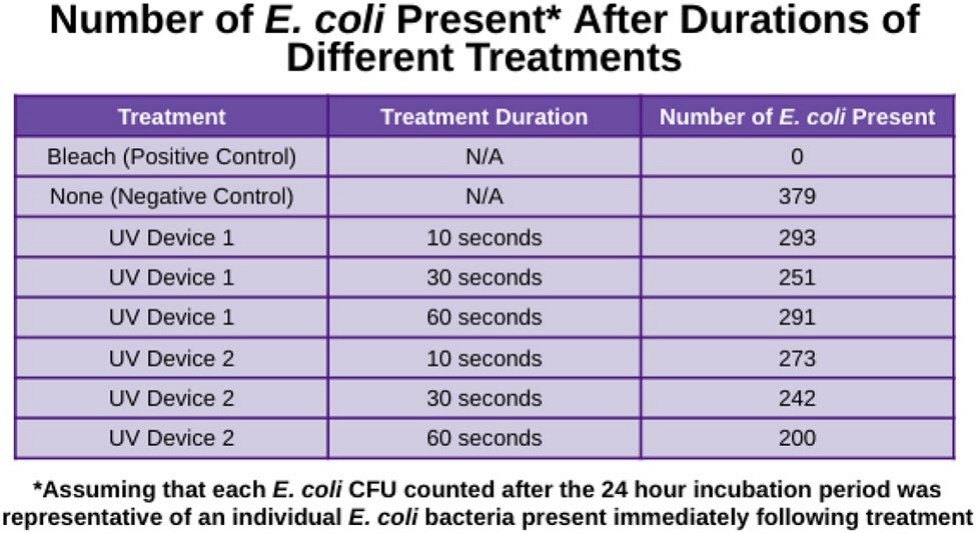

**Results:**

UV device #1 killed 23%, 34%, and 23% of E. coli at 10, 30, and 60 seconds, respectively. Similarly, device #2 killed 28%, 36%, and 47% of E. coli at 10, 30, and 60 seconds, respectively. This contradicts both companies’ claims that the UV light devices kill 99.9% in 10 seconds.

**Conclusion:**

This study showed that the kill rates of both the UV devices did not match manufacturers’ advertised claims. This may have health implications for unsuspecting consumers utilizing these devices on their food or kitchen surfaces, even after following manufacturer instructions. Further research should be conducted to verify if there is a common trend of false advertising amongst manufacturers of other handheld UV devices.

**Disclosures:**

All Authors: No reported disclosures

